# A pilot study of chemoimmunotherapy in the postconsolidation setting for high‐risk neuroblastoma (ANBL19P1): A report from the Children’s Oncology Group

**DOI:** 10.1002/cncr.70165

**Published:** 2026-01-12

**Authors:** Ami V. Desai, Arlene Naranjo, Brian LaBarre, Lulu Chen, Kelly C. Goldsmith, Meaghan P. Granger, Lisa States, Sean G. Green, Mariel Trunzo, Wendy Fitzgerald, Steven G. DuBois, Rochelle Bagatell, Julie R. Park, Araz Marachelian

**Affiliations:** ^1^ Section of Hematology, Oncology, and Stem Cell Transplantation, Department of Pediatrics, University of Chicago Chicago Illinois USA; ^2^ Children’s Oncology Group Statistics and Data Center University of Florida Gainesville Florida USA; ^3^ Aflac Cancer and Blood Disorders Center at Children’s Healthcare of Atlanta and Emory University School of Medicine Atlanta Georgia USA; ^4^ St. Jude Children's Research Hospital Memphis Tennessee USA; ^5^ Children's Hospital of Philadelphia, University of Pennsylvania Philadelphia Pennsylvania USA; ^6^ Lucile Packard Children’s Hospital Stanford Stanford California USA; ^7^ Children’s Hospital of Los Angeles Los Angeles California USA; ^8^ Dana‐Farber/Boston Children’s Cancer and Blood Disorders Center Harvard Medical School Boston Massachusetts USA; ^9^ Present address: New York‐Presbyterian Hospital New York NY USA; ^10^ Present address: United Therapeutics Corporation Research Triangle Park NC USA

**Keywords:** anti‐GD2 antibody, chemoimmunotherapy, high‐risk neuroblastoma, postconsolidation

## Abstract

**Background:**

Survival for patients with high‐risk neuroblastoma remains poor despite current‐era multimodality treatment that includes postconsolidation GD2‐directed immunotherapy. Given the promising responses in patients who receive dinutuximab‐based chemoimmunotherapy in the relapsed setting, the Children’s Oncology Group ANBL19P1 study evaluated the feasibility of administering irinotecan, temozolomide, dinutuximab, and sargramostim after frontline consolidation with tandem autologous stem cell transplantation (ASCT).

**Methods:**

Patients with high‐risk neuroblastoma who received induction therapy followed by tandem ASCT and had no evidence of progressive disease (PD) were eligible. Treatment included five 28‐day cycles of temozolomide and irinotecan (days 1–5), dinutuximab (days 2–5), and sargramostim (days 6–12). Isotretinoin (days 8–21) was given during cycles 1–6. Therapy was deemed feasible if the 95% confidence interval placed on the percentage of patients that completed five cycles of chemoimmunotherapy without PD within 30 weeks contained 75% in the absence of excessive toxicity. Event‐free survival and overall survival were determined from the time of enrollment.

**Results:**

Forty eligible patients enrolled, and 35 (87.5%; 95% confidence interval, 73.9%–94.5%) completed five cycles without PD within 30 weeks, meeting the feasibility threshold. No unacceptable toxicities occurred on protocol therapy, including no toxic deaths. Five patients discontinued therapy early because of physician determination (*n* = 2), patient/parent refusal of further therapy (*n* = 2), and PD (*n* = 1). The 2‐year event‐free and overall survival rates were 82.5% ± 6.1% and 92.5% ± 4.2%, respectively.

**Conclusions:**

The administration of chemoimmunotherapy in the postconsolidation setting after tandem ASCT is feasible and tolerable. Future studies will be needed to define the population most likely to benefit from this augmented postconsolidation therapy.

## Abstract 2

The ANBL19P1 trial demonstrated the feasibility and tolerability of administering anti‐GD2 antibody–based chemoimmunotherapy in the postconsolidation phase of frontline therapy for patients with high‐risk neuroblastoma. Survival outcomes were excellent in early follow‐up, and future studies will be needed to define the appropriate population for this augmented postconsolidation therapy.

## INTRODUCTION

Postconsolidation therapy consisting of the anti‐GD2 monoclonal antibody dinutuximab administered with the cytokines sargramostim (granulocyte‐macrophage colony‐stimulating factor [GM‐CSF]) and aldesleukin (IL‐2) and the differentiating agent isotretinoin has improved survival for newly diagnosed patients with high‐risk neuroblastoma (HR‐NBL).^1^, ^2^, ^3^ Nevertheless, approximately 25%–35% of children who complete multimodality therapy—which includes *induction* chemotherapy and surgery, *consolidation* with tandem autologous stem cell transplantation (ASCT) followed by external beam radiotherapy, and *postconsolidation* immunotherapy—experience disease recurrence.^1^, ^4^ Long‐term survival rates for those who experience a relapse post‐therapy remain poor.^4^ Novel approaches are therefore needed to eliminate minimal residual disease in the postconsolidation setting and reduce the risk of recurrence.

The Children's Oncology Group (COG) phase 2 study ANBL1221 (ClinicalTrials.gov identifier NCT01767194) demonstrated that the combination of irinotecan and temozolomide with dinutuximab and GM‐CSF is active in the relapsed/refractory disease setting, with an objective response rate (complete response [CR] and partial response [PR]) of 41.5% in the full cohort (*n* = 53).^5^, ^6^ Among those evaluable for toxicity, the most common grade 3 or higher toxicities were fever/infection (18 of 51; 35.3%), neutropenia (17 of 51; 33.3%), pain (15 of 51; 29.4%), and diarrhea (10 of 51; 19.6%). Grade 3 or higher thrombocytopenia was noted in five of 51 patients (9.8%).^6^ These data support the hypothesis that augmentation of postconsolidation immunotherapy in the frontline setting using a chemoimmunotherapy approach may be tolerable and further improve outcomes for children with HR‐NBL. However, the safety and feasibility of administering this combination shortly after tandem ASCT has not previously been investigated in a clinical trial setting. ANBL19P1 (ClinicalTrials.gov identifier NCT04385277) was designed to evaluate the feasibility and tolerability of administering dinutuximab in combination with irinotecan, temozolomide, and GM‐CSF in children with newly diagnosed HR‐NBL after induction and consolidation with myeloablative chemotherapy and tandem ASCT.

## MATERIALS AND METHODS

### Study design and participants

COG ANBL19P1 was a single‐arm pilot study of dinutuximab in combination with irinotecan, temozolomide, GM‐CSF, and isotretinoin in the postconsolidation setting. The study opened November 30, 2020, and was permanently closed to accrual June 30, 2023. The trial was approved by the National Cancer Institute Pediatric Central Institutional Review Board and local institutional review boards. Written informed consent was obtained. The study was conducted in accordance with Good Clinical Practice principles and the Declaration of Helsinki.

Eligible patients were required to be younger than 31 years at the time of enrollment and must have been designated as having HR‐NBL based on the COG risk‐classification system, including the following groups: International Neuroblastoma Risk Group Staging System (INRGSS)^7^ stage M disease with *MYCN* amplification at any age or INRGSS stage M disease in a child 18 months or older at diagnosis; INRGSS stage MS with *MYCN* amplification; or INRGSS stage L2 with *MYCN* amplification. Patients who did not undergo tandem ASCT were ineligible. Patients could not have experienced progressive disease (PD) as defined in the 2017 International Neuroblastoma Response Criteria (INRC)^8^ at any point since the initial diagnosis of HR‐NBL. As part of frontline therapy, patients must have completed four to six cycles of standard induction chemotherapy and could have received up to four cycles of postinduction or *bridging* chemotherapy or chemoimmunotherapy before consolidation provided that there was no PD. Patients were required to enroll between day +56 and day +200 from the peripheral blood stem cell infusion after completion of the last dose of high‐dose chemotherapy as part of tandem ASCT.^4^ All patients were required to have received external beam radiation therapy, unless there was both no identifiable primary tumor and no persistent metastatic disease at the end of induction. A platelet count ≥50,000/μL (transfusion‐independent for at least 7 days) was mandated. Patients with transplant‐associated thrombotic microangiopathy (TA‐TMA) were eligible if prespecified organ‐function criteria were met. Key exclusion criteria were: enrollment and treatment assignment on the concurrent COG phase 3 frontline trial ANBL1531 (ClinicalTrials.gov identifier NCT03126916); enrollment on the chemoimmunotherapy pilot study COG ANBL17P1 (ClinicalTrials.gov identifier NCT03786783); consolidation with single rather than tandem ASCT; and receipt of iodine 131‐metaiodobenzylguanidine (^131^I‐MIBG) therapy at any time before enrollment.

### Procedures

#### Treatment

During cycles 1–5, patients received intravenous irinotecan (body surface area [BSA] ≥0.6 m^2^, 50 mg/m^2^ per dose; BSA <0.6 m^2^, per protocol dosing table^9^) and oral temozolomide (BSA ≥0.6 m^2^, 100 mg/m^2^ per dose; BSA <0.6 m^2^, per protocol dosing table^9^) on days 1–5, intravenous dinutuximab (17.5 mg/m^2^ per dose) on days 2–5, subcutaneous GM‐CSF (250 mcg/m^2^ per dose on days 6–12), and oral isotretinoin (BSA ≥0.6 m^2^, 80 mg/m^2^ per dose twice daily; BSA <0.6 m^2^, per protocol dosing table^9^) on days 8–21. Cycle 6 consisted of isotretinoin (BSA ≥0.6 m^2^, 80 mg/m^2^ per dose twice daily; BSA <0.6 m^2^, per protocol dosing table^9^) alone on days 8–21. Although chemoimmunotherapy in the relapsed/refractory setting was administered every 21 days on ANBL1221,^5^, ^6^ for the current trial, we used 28‐day cycles to align with standard postconsolidation dinutuximab administration on ANBL0032.^1^ A 28‐day cycle also permitted a 7‐day window between the last dose of isotretinoin and the start of the next cycle of chemotherapy. Dose reduction or omission of isotretinoin was permitted for patients with TA‐TMA based on institutional guidelines.

#### Assessment of toxicity and disease evaluations

Toxicity was assessed using the Common Terminology Criteria for Adverse Events version 5.0. Disease evaluations, including computed tomography (CT) or magnetic resonance imaging (MRI) tumor imaging, a ^123^I‐MIBG scan or fludeoxyglucose‐18–positron emission tomography (^18^FDG‐PET) scan for patients with MIBG non‐avid disease at diagnosis, and bilateral bone marrow aspirates and trephine biopsies, were performed within 21 days of study enrollment and at the end of protocol therapy (defined as after cycle 6 or after cycle 5 for patients who discontinued isotretinoin early). A ^123^I‐MIBG scan (or ^18^FDG‐PET scan, if applicable) was required for all patients after cycle 3. However, bone marrow studies were not required at this timepoint if no bone marrow involvement was detected at enrollment on ANBL19P1, and CT/MRI imaging was optional for patients with no residual soft tissue disease at enrollment. During follow‐up, CT or MRI studies were required at 6, 12, 18, 24, and 36 months, and ^123^I‐MIBG scans (or ^18^FDG‐PET scans, if applicable) were required at 3, 6, 12, 18, 24, and 36 months. Full disease evaluations were obtained at relapse.

### Statistical analyses

#### Determination of feasibility

The primary objective of this study was to determine the feasibility of administering five cycles of chemoimmunotherapy without PD within 30 weeks from the date of first treatment among patients who started protocol therapy. This was assessed by estimating the therapy completion rate together with a 95% Wilson confidence interval (CI). The therapy would be deemed feasible if the 95% CI placed on the percentage of patients who completed at least five cycles of dinutuximab plus chemotherapy without PD within 30 weeks contained 75%, and if the interim monitoring rules for feasibility and excessive toxicity were not triggered. With 40 eligible and evaluable patients, the estimate of feasibility would have a standard error of 0.0791 at most.

The feasibility monitoring rule assessed whether ≥75% of patients who started protocol therapy completed at least three cycles of dinutuximab + chemotherapy without PD within 18 weeks. Unacceptable toxicities were defined as those standard for dinutuximab‐based protocols (use of pressors for ≥24 hours, including those associated with grade 4 capillary leak syndrome, grade 4 anaphylaxis/allergic reaction, or grade 3 or 4 hypotension; ventilatory support ≥24 hours, including grade 4 respiratory toxicity, such as acute respiratory distress syndrome, grade 4 bronchospasm, grade 4 dyspnea, grade 4 hypoxia, grade 4 anaphylaxis/allergic reaction, or grade 4 respiratory failure requiring intubation and mechanical ventilation; and grade 3 or 4 peripheral motor or sensory neuropathy that did not resolve before the start of the next cycle) as well as Common Terminology Criteria for Adverse Events version 5.0 grade 4 documented infection and grade 4 diarrhea that persisted despite recommended supportive care. An unacceptable toxicity rate statistically significantly greater than 10% was considered excessive on this trial.

Patients who had dose modifications but remained on protocol therapy and completed all five chemoimmunotherapy cycles within the specified timeframe were not counted against feasibility. Discontinuation of isotretinoin also did not count against feasibility. Any eligible patient who received at least one dose of protocol therapy was considered evaluable for the primary endpoint.

If neutropenia or thrombocytopenia resulted in a delay of ≥14 days between treatment cycles, the temozolomide dose was reduced by 25% for subsequent cycles. If this delay recurred despite the dose modification, the patient was taken off protocol therapy. If delayed recovery of platelets and neutrophils resulted in a ≥28‐day delay in starting the next cycle, the patient was taken off protocol therapy. For non‐hematologic toxicity, including diarrhea, nausea/vomiting, dehydration, transaminitis, or elevated γ‐glutamyl transferase, protocol‐specific guidelines for dose modification of chemotherapy and/or dinutuximab or discontinuation of protocol therapy were followed as detailed in Table S1. For all other grade 3 or higher non‐hematologic toxicities or hematologic toxicities that prolonged the start of subsequent cycles by ≥14 days, both irinotecan and temozolomide were dose‐reduced by 25% in the subsequent cycle. If the toxicity recurred with a delay of ≥14 days, the patient was removed from protocol therapy.

#### Demographics, disease characteristics, prior therapy

Demographics, clinical characteristics, tumor characteristics (including *MYCN* status, histology, and ploidy), relevant medical history (e.g., TA‐TMA), prior therapy, and 2017 INRC response^8^ at enrollment compared with disease status at diagnosis were summarized using descriptive statistics.

#### Toxicities

All eligible patients who received at least one dose of irinotecan or temozolomide were considered evaluable for toxicity. Grade 3 or higher adverse events were captured and summarized descriptively.

#### Response assessment and outcomes

All eligible patients with evaluable or measurable disease at study entry who received at least one dose of irinotecan or temozolomide were considered evaluable for response. Response at each disease evaluation timepoint was compared with disease status at time of enrollment as assessed according to the 2017 INRC.^8^

Event‐free survival (EFS) and overall survival (OS) were determined from the time of enrollment. Survival estimates were generated using Kaplan–Meier curves.

## RESULTS

### Patient characteristics

**FIGURE 1:**
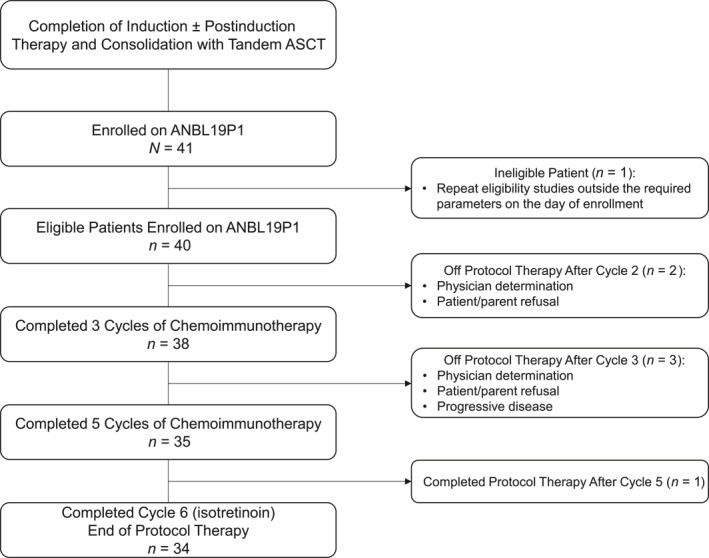
The ANBL19P1 cohort. Forty‐one patients enrolled and 40 eligible patients began protocol therapy. ASCT indicates autologous stem cell transplantation.

Forty‐one patients enrolled, and 40 eligible patients started protocol therapy (Figure 1). One patient was deemed ineligible because repeat eligibility studies were outside the required parameters on the day of enrollment. Patient characteristics are summarized in Table 1. The majority were ≥18 months of age at high‐risk diagnosis (*n* = 35; 87.5%) and had INRGSS stage M disease (*n* = 39; 97.5%). Among those with known tumor biology, 44.7% (*n* = 17 of 38) had tumors that were *MYCN*‐amplified, 97.4% (*n* = 37 of 38) had tumors with unfavorable histology, and 91.7% (*n* = 11 of 12) had diploid tumors. Among those with available race and ethnicity data, most were White (78.8%; 26 of 33) and non‐Hispanic/Latino (72.2%; 26 of 36).

**TABLE 1:** Cohort description.

Characteristic	ANBL19P1 participants, *N* = 40
Age at high‐risk diagnosis, No. (%)	
<18 months	5 (12.5)
≥18 months	35 (87.5)
INRGSS stage at diagnosis,^a^ No. (%)	
M	39 (97.5)
MS	0 (0.0)
L2	1 (2.5)
*MYCN* status, No. (%)	
Not amplified	21 (55.3)
Amplified	17 (44.7)
Unknown	2
Histology, No. (%)	
Favorable	1 (2.6)
Unfavorable	37 (97.4)
Unknown	2
Ploidy, No. (%)	
Hyperdiploid	1 (8.3)
Diploid	11 (91.7)
Unknown	28
Time from last ASCT to enrollment: Median [range], days	90.5 [70–194]
Received postinduction/bridging chemotherapy or chemoimmunotherapy, No. (%)	
Yes^b^	6 (15.0)
No	34 (85.0)
Prior anti‐GD2 therapy, No. (%)	
Yes	9 (22.5)
No	31 (77.5)
INRC response at enrollment (compared to disease status at diagnosis), No. (%)	
Complete response	21 (52.5)
Partial response	16 (40.0)
Minor response	3 (7.5)
Stable disease	0 (0.0)
Sites of disease at enrollment,^c^ No. (%)	
Primary tumor/locoregional disease	14 (35.0)
Metastatic soft tissue	1 (2.5)
Bone	8 (20.0)
Bone marrow	3 (7.5)
None	21 (52.5)
Race, No. (%)	
American Indian or Alaska Native	0 (0.0)
Asian	3 (9.1)
Native Hawaiian or other Pacific Islander	1 (3.0)
Black or African American	2 (6.1)
White	26 (78.8)
More than one race	1 (3.0)
Unknown or not reported	7
Ethnicity, No. (%)	
Hispanic or Latino	10 (27.8)
Not Hispanic or Latino	26 (72.2)
Unknown or not reported	4
Sex, No. (%)	
Female	20 (50.0)
Male	20 (50.0)

Abbreviations: ASCT, autologous stem cell transplantation; GD2, disialoganglioside GD2; INRC, International Neuroblastoam Response Criteria; INRGSS, International Neuroblastoma Risk Group Staging System.

^a^ L2 indicates locoregional tumor with the presence of one or more image‐defined risk factor(s); M, distant metastatic disease; MS, metastatic disease in children younger than 18 months with metastases confined to skin, liver, and/or bone marrow (≤10%).

^b^ All patients who received postinduction/bridging therapy received chemoimmunotherapy.

^c^ Patients may have disease involvement at one or more sites at enrollment.

Nine patients (22.5%) had received anti‐GD2 therapy at any time before enrollment. Of these, six (15.0%) had received postinduction/bridging chemoimmunotherapy with dinutuximab, irinotecan, and temozolomide (Table 1). Three had received anti‐GD2 antibody concurrently with five or six cycles of standard North American induction chemotherapy (cyclophosphamide/topotecan, cisplatin/etoposide, vincristine/doxorubicin/cyclophosphamide) according to institutional practice. All patients had received external beam radiotherapy as part of consolidation. At enrollment, approximately one half of the cohort (52.5%) were in a CR. The most common site of residual disease at enrollment was the primary tumor site and/or locoregional disease (*n* = 14; 35.0%) followed by bone (*n* = 8; 20.0%). One patient had a history of TA‐TMA.

### Assessment of feasibility

Administration of chemoimmunotherapy in postconsolidation was feasible. Thirty‐five of 40 patients (87.5%; 95% CI, 73.9%–94.5%) completed five cycles of dinutuximab plus chemotherapy without PD within 30 weeks, meeting the feasibility benchmark of 75%. The feasibility and excessive toxicity monitoring rules were not triggered, and no unacceptable toxicities were observed. Reasons for early discontinuation of protocol therapy, all of which occurred before cycle 4, included physician determination (*n* = 2), patient/parent refusal of further therapy (*n* = 2), and PD (*n* = 1), as summarized in Figure 1. For cycles 1–5, the median cycle length was 28 days (range, 23–57 days).

### Treatment delays and dose modifications

#### Treatment delays

Treatment delays occurred in 11 patients (27.5%), affecting 18 of 222 (8.1%) total cycles delivered. Five patients had delays secondary to non‐hematologic toxicity: specifically, infection (*n* = 3) and elevated liver enzymes (*n* = 2). Hematologic toxicity led to treatment delays in five patients who experienced neutropenia (six cycles) and/or thrombocytopenia (12 cycles). Other documented reasons for treatment delays were holiday schedule (*n* = 1), issue with the Risk Evaluation and Management Strategy safety program required by the US Food and Drug Administration for dispensing of isotretinoin (*n* = 1), and parental decision (*n* = 1).

#### Dose modifications

Treatment dose modifications, including dose holds and dose reductions, are summarized (Table 2). Dose modification of any agent occurred in nine patients (22.5%), which affected 5.4% (12/222) total cycles delivered. Irinotecan, temozolomide, and dinutuximab were all held in one patient who had a seizure of unclear etiology; the same patient had dinutuximab held in the following cycle for rigors. Subsequently, this patient was taken off protocol therapy by patient/parent request. Three other patients had a dose reduction in temozolomide for hematologic toxicity. Two additional patients underwent dose reduction of dinutuximab according toxicity management guidelines. Isotretinoin was held for proteinuria in one patient. Another patient had missed doses because of a system issue with the isotretinoin Risk Evaluation and Management Strategy safety program in one cycle and an unplanned dose reduction in a later cycle because of a change in personality. Two additional patients had missed doses of isotretinoin because of poor adherence.

**TABLE 2:** Treatment dose modifications.

Agent	No. (%) of patients with dose modifications, *N* = 40	No. (%) of cycles with dose modifications, *N* = 222 total cycles
Any agent	9 (22.5)	12 (5.4)
Irinotecan	1 (2.5)	1 (0.5)
Temozolomide	4 (10.0)	4 (1.8)
Dinutuximab	3 (7.5)	4 (1.8)
Isotretinoin	4 (10.0)	6 (2.7)

### Toxicities

Treatment‐related grade 3 adverse events and grade 4 or 5 adverse events of any attribution with a frequency ≥5% at any time and by cycle are summarized in Table 3. Seven of 40 patients (17.5%) did not have an adverse event that met the aforementioned grade and frequency criteria at any time. Fever (*n* = 11; 27.5%) and pain (*n* = 11; 27.5%) were most common in cycle 1 and decreased in frequency during subsequent cycles. Infectious adverse events, such as enterocolitis and sepsis, were infrequent and occurred in <10% of patients during cycle 2 and/or cycle 3. Elevation of liver enzymes was more common during later cycles. Hematologic adverse events, such as low neutrophil count (*n* = 10; 25.0%) and lymphocyte count (*n* = 9; 22.5%), were most frequently noted in cycle 1 and generally improved over the course of therapy. Anorexia and dehydration were rare events and were observed in earlier cycles. Grade 3 or higher diarrhea was also rare, occurring cumulatively in 10.0% of patients (*n* = 4). Similarly, bronchospasm and hypotension were rare events and were only noted in 5.0% (*n* = 2) and 7.5% (*n* = 3) of patients at any time, respectively. Hypoxia and hypertension were each noted in 10.0% of patients (*n* = 4) at any time. Hypokalemia was the most frequently noted electrolyte abnormality (42.5% of patients at any time; *n* = 17).

**TABLE 3:** Patients with treatment‐related adverse events grade ≥3 (possible, probable, definite), grade 4 (any attribution), and grade 5 (any attribution) ≥5% at any time.

	No. (%)						
CTCAE v5.0 adverse event	Any cycle, *N* = 40	Cycle 1, *N* = 40	Cycle 2, *N* = 40	Cycle 3, *N* = 38	Cycle 4, *N* = 35	Cycle 5, *N* = 35	Cycle 6, *N* = 34
None	7 (17.5)	13 (32.5)	17 (42.5)	15 (39.5)	19 (54.3)	20 (57.1)	32 (94.1)
Pain	13 (32.5)	11 (27.5)	5 (12.5)	3 (7.9)	3 (8.6)	1 (2.9)	—
Febrile neutropenia	7 (17.5)	3 (7.5)	2 (5.0)	2 (5.3)	2 (5.7)	1 (2.9)	—
Fever	17 (42.5)	11 (27.5)	5 (12.5)	7 (18.4)	6 (17.1)	3 (8.6)	—
Enterocolitis infectious	3 (7.5)	—	3 (7.5)	—	—	—	—
Sepsis	6 (15.0)	—	2 (5.0)	3 (7.9)	1 (2.9)	1 (2.9)	—
Lymphocyte count decreased	12 (30.0)	9 (22.5)	8 (20.0)	3 (7.9)	3 (8.6)	4 (11.4)	—
Neutrophil count decreased	12 (30.0)	10 (25.0)	3 (7.5)	2 (5.3)	2 (5.7)	2 (5.7)	—
Platelet count decreased	8 (20.0)	5 (12.5)	1 (2.5)	3 (7.9)	2 (5.7)	2 (5.7)	—
White blood cell decreased	9 (22.5)	5 (12.5)	3 (7.5)	1 (2.6)	3 (8.6)	2 (5.7)	—
Alanine aminotransferase increased	6 (15.0)	2 (5.0)	2 (5.0)	4 (10.5)	2 (5.7)	4 (11.4)	—
Aspartate aminotransferase increased	4 (10.0)	2 (5.0)	1 (2.5)	2 (5.3)	3 (8.6)	3 (8.6)	—
GGT increased	4 (10.0)	4 (10.0)	—	—	—	—	—
Anorexia	4 (10.0)	2 (5.0)	1 (2.5)	3 (7.9)	1 (2.9)	—	—
Dehydration	3 (7.5)	2 (5.0)	1 (2.5)	—	—	—	—
Diarrhea	4 (10.0)	2 (5.0)	2 (5.0)	—	1 (2.9)	2 (5.7)	—
Bronchospasm	2 (5.0)	2 (5.0)	—	—	—	—	—
Hypoxia	4 (10.0)	1 (2.5)	1 (2.5)	1 (2.6)	2 (5.7)	—	—
Hypotension	3 (7.5)	2 (5.0)	‐	1 (2.6)	1 (2.9)	—	—
Hypertension	4 (10.0)	2 (5.0)	2 (5.0)	2 (5.3)	—	1 (2.9)	—
Hypokalemia	17 (42.5)	8 (20.0)	5 (12.5)	3 (7.9)	6 (17.1)	1 (2.9)	—

Abbreviations: CTCAE v5.0, Common Terminology Criteria for Adverse Event, version 5.0; GGT, γ‐glutamyl transferase.

### Disease response

Twenty‐one patients entered the study in CR, and all remained without evidence of disease at the time of discontinuation of protocol therapy. Among the 19 patients who entered the study with a response of less than CR, 11 had only measurable disease at the primary tumor site/locoregional disease, and eight had metastatic disease. Sixteen of these 19 patients had a full evaluation at the time of discontinuation of protocol therapy. The objective response rate among the 16 patients who were not in CR at study entry and had a full evaluation at therapy discontinuation was 31.3% (PR, *n* = 4; minor response [MR], *n* = 1). In addition, eight patients had stable disease, of whom six had disease limited to the primary tumor site at study entry. The disease control rate (PR/MR/stable disease) within this group was 81.3% (13 of 16 patients). Three of 16 patients (18.8%) had PD. Among the 19 patients who were not fully evaluated for response (*n* = 3), one with bone‐only metastatic disease at study entry (Curie score = 8) had resolution of all bony sites. However, the overall response was recorded as not done according to INRC^8^ because bone marrow evaluation was not completed.

### Survival outcomes

The 2‐year EFS and OS of the full cohort (*n* = 40) were 82.5% ± 6.1% and 92.5% ± 4.2%, respectively (Figure 2A). The median follow‐up time for 31 patients without an event was 2.8 years (range, 2.0–3.8 years).

FIGURE 2Event‐free survival and overall survival for the entire cohort and by disease status. (A) EFS and OS for the entire cohort (n = 40), (B) EFS by disease status at enrollment (CR vs. PR/MR (i.e. non‐CR); *p* = .0004), (C) OS by disease status at enrollment (CR vs. PR/MR (i.e. non‐CR); *p* = .0612), (D) EFS by metastatic bone or bone marrow status at enrollment (*p* = .0012), and (E) OS by metastatic bone or bone marrow status at enrollment (*p* = .5189). CR indicates complete response; EFS, event‐free survival; MR, minor response; OS, overall survival; PR, partial response; SE, standard error.

**Figure:**
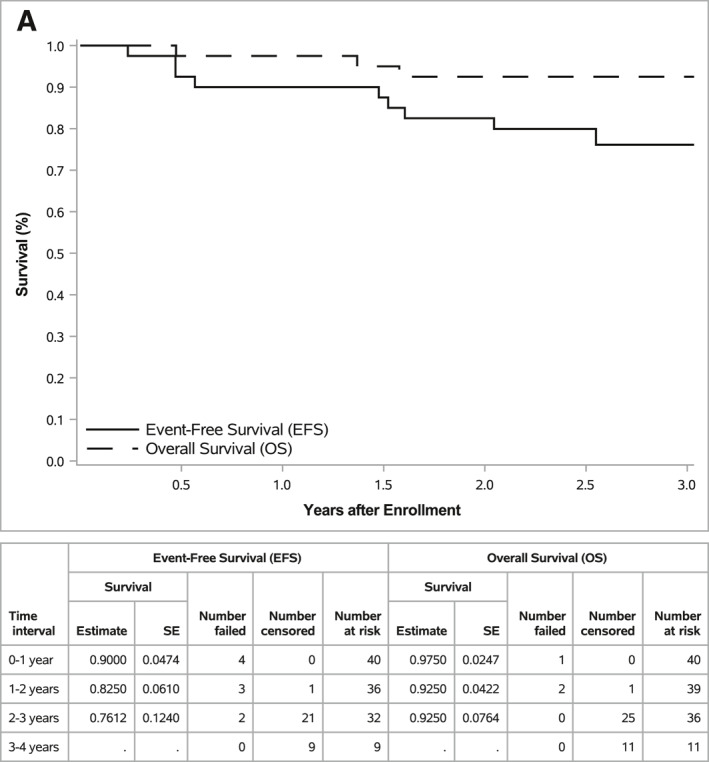

**Figure:**
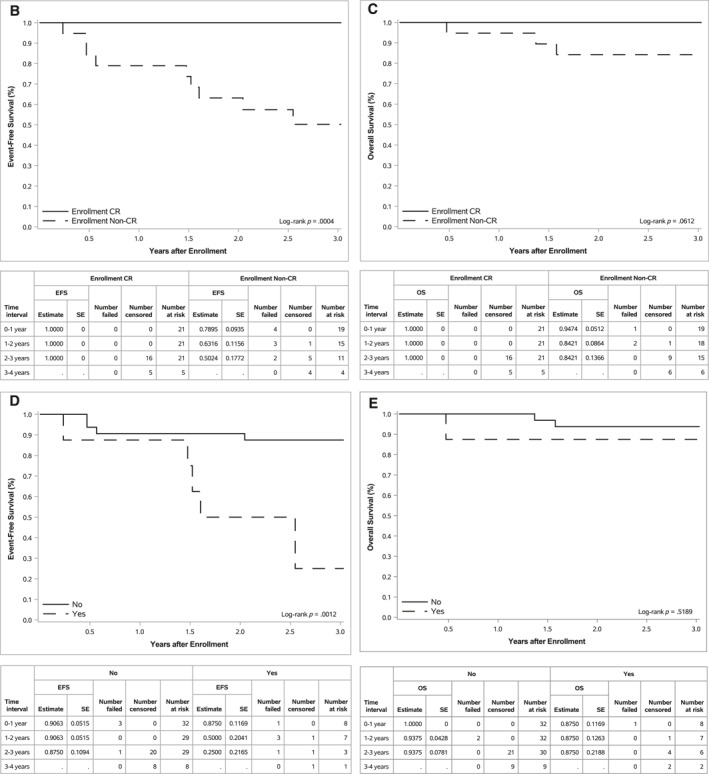

EFS and OS by disease status at enrollment (i.e., response after consolidation compared with diagnosis), presence of metastatic bone or bone marrow disease at enrollment, receipt of postinduction/bridging therapy, and completion of five cycles of chemoimmunotherapy are summarized in Table 4. Patients who entered the study in CR had superior EFS compared with those who entered the study with less than a CR (*p* = .0004; Figure 2B), with a trend toward improved OS (*p* = .0612; Figure 2C). Because small amounts of residual treated soft tissue remaining at the primary tumor site can frequently result in an overall response of PR according to INRC^8^ at the end of consolidation, the effect of bone marrow and cortical bony disease at enrollment was also specifically evaluated. EFS was significantly lower among patients who had metastatic bone/bone marrow disease at enrollment compared with the EFS of patients who had no metastatic disease at those sites (*p* = .0012; Figure 2D); however, OS did not differ (*p* = .5189; Figure 2E). Patients who had received additional postinduction/bridging therapy before consolidation also had inferior EFS (*p* = .0012), but not inferior OS (*p* = .3546), compared with those who only received standard induction therapy. EFS and OS were no different among those who completed five cycles of chemoimmunotherapy compared with those who completed less than five cycles of chemoimmunotherapy on study (EFS, *p* = .9210; OS, *p* = .2198).

**TABLE 4:** Event‐free survival and overall survival by patient characteristics.

Patient characteristic	No. (%)	2‐year EFS ± SE, %	*p*	2‐year OS ± SE, %	*p*
INRC response at enrollment compared to initial diagnosis of high‐risk neuroblastoma					
CR	21 (52.5)	100.0 ± 0.0	.0004^a^	100.0 ± 0.0	.0612^a^
PR	16 (40.0)	62.5 ± 12.8		81.3 ± 10.2	
MR	3 (7.5)	66.7 ± 27.2		100.0 ± 0.0	
Metastatic bone or bone marrow present at enrollment					
Yes	8 (20.0)	50.0 ± 20.4	.0012	87.5 ± 12.6	.5189
No	32 (80.0)	90.6 ± 5.2		93.8 ± 4.3	
Received postinduction/bridging therapy					
Yes	6 (15.0)	33.3 ± 19.3	.0012	83.3 ± 15.2	.3546
No	34 (85.0)	91.2 ± 4.9		94.1 ± 4.1	
No. of cycles of ANBL19P1 protocol therapy completed					
5	35 (87.5)	82.9 ± 6.5	.9210	94.3 ± 4.0	.2198
<5	5 (12.5)	80.0 ± 17.9		80.0 ± 17.9	

Abbreviations: CR, complete response; EFS, event‐free survival; INRC, International Neuroblastoma Response Criteria; MR, minor response; OS, overall survival; PR, partial response; SE, standard error.

^a^ CR versus PR/MR.

## DISCUSSION

ANBL19P1 is the first trial to demonstrate the feasibility and tolerability of administering chemoimmunotherapy after tandem ASCT in patients with HR‐NBL. Importantly, no unacceptable toxicities occurred within this cohort, particularly grade 4 infection and grade 4 diarrhea, with the addition of chemotherapy in this setting. Hematologic adverse events were less frequent over the course of five cycles, and only three patients had a chemotherapy dose reduction because of cytopenias, further supporting the tolerability of administration of this regimen in postconsolidation. Pain was most frequent during cycle 1 and generally improved over subsequent cycles; this pattern is similar to that observed in patients who receive treatment with standard immunotherapy.^1^, ^3^

In early follow‐up, the EFS and OS of the full cohort were excellent, with 2‐year EFS and OS of 82.5% and 92.5% from the time of enrollment, respectively. The 2‐year EFS and OS of patients randomized to postconsolidation immunotherapy with dinutuximab and cytokines on ANBL0032 were 66% and 86%, respectively.^1^ Similarly, among those who received immunotherapy on ANBL0032 postrandomization, the 2‐year EFS and OS were 69.4% and 84%, respectively.^3^ Although a direct comparison cannot be made between survival outcomes on ANBL0032 because of cohort differences (e.g., the number of ASCTs administered and disease status preconsolidation and at the time of enrollment), the excellent 2‐year EFS and OS on ANBL19P1 are promising. EFS was superior among patients who had a CR compared with those who had less than a CR at the time of enrollment. This is consistent with the experience reported among patients who received standard postconsolidation immunotherapy after the cessation of randomization on ANBL0032.^3^ Longer follow‐up of a larger cohort of patients will be helpful to determine whether the excellent survival outcomes observed, particularly in patients with a CR after consolidation, are sustained over time.

Only eight patients (20%) had metastatic disease at study entry and, within this group, two patients achieved a PR, one had a MR, and one had a CR in the metastatic compartment (all bone disease) according to the 2017 INRC.^8^ The receipt of external beam radiotherapy to the primary tumor site and metastatic lesions during consolidation, just before enrollment, should be taken into consideration when interpreting disease response in this trial. Additional follow‐up of the current cohort and evaluation of this postconsolidation regimen in a larger group of patients with residual metastatic disease are needed to determine whether chemoimmunotherapy late in the course of treatment can improve survival outcomes. This question is particularly relevant because chemoimmunotherapy is now being administered earlier in frontline therapy^10^, ^11^, ^12^, ^13^, ^14^ and is widely used as bridging therapy for patients who have a less than optimal response before consolidation.^15^

As the role of GD2‐directed therapy continues to expand, so too does interest in identifying biomarkers that predict treatment response and survival. Recent efforts have focused on both tumor‐intrinsic and immune‐related biomarkers.^16^ Although the disialoganglioside GD2 is structurally complex^17^ and has been challenging to measure to date, promising approaches to quantify GD2 expression include analysis of circulating GD2 shed from tumor,^18^, ^19^, ^20^, ^21^ tumor cell–bound GD2 in bone marrow,^22^ immunofluorescence staining of formalin‐fixed paraffin‐embedded tissue,^23^ all of which have the potential to be scalable, predictive biomarkers. Among immune‐related biomarkers investigated to date, KIR (killer cell immunoglobulin‐like receptor)/KIR‐ligand genotype status was associated with outcomes in a cohort of patients receiving postconsolidation with anti‐GD2 antibody^24^ as well as in a cohort of patients receiving anti‐GD2–based chemoimmunotherapy^25^; the potential to use KIR/KIR‐ligand status as a screening biomarker warrants further investigation. FcγR3A (Fc gamma receptor IIIa) has been associated with improved outcomes in some,^3^, ^26^ but not all,^2^, ^25^ cohorts receiving anti‐GD2–based antibody therapy. Comprehensive profiling of the tumor immune microenvironment^27^ and peripheral immune repertoire^16^ provides insights into tumor and host characteristics that can potentially aid in the selection of patients most likely to benefit from GD2‐directed therapy. Correlative studies from ANBL19P1, including immunophenotyping and cytokine analysis as well as circulating GD2 quantification, are ongoing.

## CONCLUSION

The ANBL19P1 trial has demonstrated the feasibility of administering chemoimmunotherapy in the postconsolidation setting after undergoing tandem ASCT and has shown that this therapy is tolerable. This chemoimmunotherapy regimen may benefit patients with residual disease after consolidation therapy, and further study in a cohort larger than that included in this pilot study is needed. Rigorous evaluation of candidate biomarkers may help identify patients most likely to benefit from this therapy.

## AUTHOR CONTRIBUTIONS

**Ami V. Desai**: Conceptualization, writing–original draft, writing–review and editing, data curation, and supervision. **Arlene Naranjo**: Conceptualization, data curation, supervision, formal analysis, and writing–review and editing. **Brian LaBarre**: Formal analysis, Data curation, and writing–review and editing. **Lulu Chen**: Writing–review and editing, formal analysis, and data curation. **Kelly C. Goldsmith**: Conceptualization and writing–review & editing. **Meaghan P. Granger**: Writing–review and editing. **Lisa States**: Writing–review and editing. **Sean G. Green**: Writing–review and editing. **Mariel Trunzo**: Writing–review and editing. **Wendy Fitzgerald**: Writing–review and editing. **Steven G. DuBois**: Writing–review and editing, conceptualization, and supervision. **Rochelle Bagatell**: Conceptualization, writing–review & editing, and supervision. **Julie R. Park**: Conceptualization, writing–review and editing, and supervision. **Araz Marachelian**: Conceptualization, writing–original draft, data curation, supervision, and writing–review and editing.

## CONFLICT OF INTEREST STATEMENT

Ami V. Desai reports personal/consulting or advisory board fees from Recordati Rare Diseases Inc. and stock ownership in Pfizer and Viatris outside the submitted work. Arlene Naranjo serves on a data safety and monitoring committee for Novartis Pharma AG outside the submitted work. Kelly C. Goldsmith reports noncompensated membership on advisory boards for AbbVie and Y‐Mab outside the submitted work. Steven G. DuBois reports grants/contracts from Alex’s Lemonade Stand and advisory board fees from EMD Serono, InhibRx, and Merck outside the submitted work. Rochelle Bagatell reports personal/consulting fees from Merck outside the submitted work. Araz Marachelian reports grants/contracts from Recordati Rare Diseases Inc. and United Therapeutics Corporation outside the submitted work. The remaining authors disclosed no conflicts of interest.

## Supporting information

Table S1

## Data Availability

Requests for access to Children's Oncology Group protocol research data should be sent to: datarequest@childrensoncologygroup.org.
