# Supplementary material for: A pilot study of chemoimmunotherapy in the postconsolidation setting for high‐risk neuroblastoma (ANBL19P1): A report from the Children’s Oncology Group
Source: Cancer. 2026 Jan 12;132(2):e70165. doi: 10.1002/cncr.70165 (PMC12796553; doi:10.1002/cncr.70165)
Supplement: Supplementary file 1 — Table S1 [file CNCR-132-e70165-s001.docx]

**TABLE S1: Chemoimmunotherapy Dose Modifications for Non-Hematologic Toxicities**

| Grade 4 therapy-associated diarrhea despite maximal supportive care mandated the following | - 25% dose reduction of irinotecan for subsequent cycles - 25% dose reduction in dinutuximab if the Grade 4 diarrhea persisted despite supportive care and the dose reduction in irinotecan - Off protocol therapy if Grade 4 diarrhea persisted despite supportive care and the above two dose reductions. |
| --- | --- |
| Severe nausea and vomiting, defined as Grade 4 regimen-related or Grade 3 regimen-related > 7 days | - Adjustment in the antiemetic regimen in the next cycle of therapy if inadequate in the prior cycle - 25% dose reduction in irinotecan and temozolomide for subsequent cycles if severe nausea and vomiting persisted despite optimized antiemetic plan - Off protocol therapy if severe nausea vomiting persisted despite the dose reduction in both agents |
| Dehydration | - If dehydration was related to diarrhea or nausea/vomiting, the above-mentioned toxicity guidelines were followed. - If treatment-related Grade ≥3 persisted for >3 days in the absence of significant diarrhea or nausea/vomiting, irinotecan and temozolomide were dose reduced by 25% in subsequent cycles. - If Grade ≥3 treatment-related dehydration recurred and persisted for >3 days despite the dose reductions, the patient was taken off protocol therapy. |
| Elevations in ALT, AST, or GGT, >20 x upper limit of normal (ULN) or >10x ULN to < 20x ULN and persisted for >7 days | - Dose of dinutuximab reduced by 25% for subsequent cycles (ALT ULN=45 U/L for the study). - If the elevation in the same liver enzyme recurs despite this dose reduction, temozolomide was reduced by 25% for subsequent cycles. - If the elevation in liver enzyme recurs despite these two dose reductions, the patient was taken off protocol therapy. - An elevation in ALT that causes a delay of ≥14 days between treatment cycles, the dose of dinutuximab was reduced by 25% for subsequent cycles. - If the elevation in ALT recurred, the patient was taken off protocol therapy. |

*Additional guidance on dose modification and/or toxicity management of dinutuximab, sargramostim, and isotretinoin was provided in the protocol.
